# The Role of Sarcosine, Uracil, and Kynurenic Acid Metabolism in Urine for Diagnosis and Progression Monitoring of Prostate Cancer

**DOI:** 10.3390/metabo7010009

**Published:** 2017-02-23

**Authors:** Georgios Gkotsos, Christina Virgiliou, Ioanna Lagoudaki, Chrysanthi Sardeli, Nikolaos Raikos, Georgios Theodoridis, Georgios Dimitriadis

**Affiliations:** 1 Department of Urology, School of Medicine, Faculty of Health Sciences, Aristotle University of Thessaloniki, 54124 Thessaloniki, Greece; ggkotsos@gmail.com (G.G.); gdimit@auth.gr (G.D.); 2 Department of Chemistry, Aristotle University of Thessaloniki, 54124 Thessaloniki, Greece; joanna_lagou@yahoo.gr (I.L.); gtheodor@chem.auth.gr (G.T.); 3 Department of Pharmacology & Clinical Pharmacology, School of Medicine, Faculty of Health Sciences, Aristotle University of Thessaloniki, 54124 Thessaloniki, Greece; 4 Department of Forensic Medicine & Toxicology, School of Medicine, Faculty of Health Sciences, Aristotle University of Thessaloniki, 54124 Thessaloniki, Greece; raikos@auth.gr

**Keywords:** prostate cancer, prostate cancer detection and progression, metabolite profiling, metabolomics, sarcosine, uracil, kynurenic acid

## Abstract

The aim of this pilot study is to evaluate sarcosine, uracil, and kynurenic acid in urine as potential biomarkers in prostate cancer detection and progression monitoring. Sarcosine, uracil, and kynurenic acid were measured in urine samples of 32 prostate cancer patients prior to radical prostatectomy, 101 patients with increased prostate-specific antigen prior to ultrasonographically-guided prostatic biopsy collected before and after prostatic massage, and 15 healthy volunteers (controls). The results were related to histopathologic data, Gleason score, and PSA (Prostate Specific Antigen). Metabolites were measured after analysis of urine samples with Ultra-High Performance Liquid Chromatography coupled to tandem mass spectrometry (UPLC-MS/MS) instrumentation. Multivariate, nonparametric statistical tests including receiver operating characteristics analyses, one-way analysis of variance (Kruskal–Wallis test), parametric statistical analysis, and Pearson correlation, were performed to evaluate diagnostic performance. Decreased median sarcosine and kynurenic acid and increased uracil concentrations were observed for patients with prostate cancer compared to participants without malignancy. Results showed that there was no correlation between the concentration of the studied metabolites and the cancer grade (Gleason score <7 vs. ≥7) and the age of the patients. Evaluation of biomarkers by ROC (Receiving Operating Characteristics) curve analysis showed that differentiation of prostate cancer patients from participants without malignancy was not enhanced by sarcosine or uracil levels in urine. In contrast to total PSA values, kynurenic acid was found a promising biomarker for the detection of prostate cancer particularly in cases where collection of urine samples was performed after prostatic massage. Sarcosine and uracil in urine samples of patients with prostate cancer were not found as significant biomarkers for the diagnosis of prostate cancer. None of the three metabolites can be used reliably for monitoring the progress of the disease.

## 1. Introduction

Identification and quantification of numerous endogenous metabolites in various complex biological samples became possible due to recent advances in mass spectrometry-related technologies. Results of such analysis may provide a better understanding of alterations in molecular pathways of patients including those with cancer [1,2]. Recent studies on metabolic profiling of tissues, blood, and urine samples from patients with prostate cancer (PCa) support this approach. Data shows that metabolomics may help improving diagnosis and provide information with regards to tumor progression and invasion patterns by providing detailed read-outs of tumor cell physiology and biochemical activity making [3,4,5]. Metabolic profiling of samples from PCa patients is of great interest because it may lead to the detection and identification of statistical significant biomarkers related to prostate cancer and subsequently solve the problem of low specificity and sensitivity of the current PCa screening, diagnosis, staging, and monitoring protocols based on measurements of the serum prostate-specific antigen (PSA) or its derivatives, digital rectal examination (DRE), histopathological studies of core needle biopsies, and Gleason Scores [6,7,8,9,10,11].

Regarding prostate cancer, sarcosine was initially reported as the most promising biomarker of cancer cell invasion and aggressivity [5] although its significance has been doubted by newer data [11,12]. Urinary, plasma, and tissue kynurenic acid and uracil were also found to possibly correlate with PCa progression [5]. Recently, it was found [13] that kynurenic acid in prostatic tissue may correlate with aggressiveness of prostate cancer as that was measured by Gleason scores. In a previous study, reported data showed that kynurenine and uracil in patients with genitourinary malignancies were not reliable biomarkers for the diagnosis of the disease or to predict tumor aggressiveness [14,15]. The aim of this study is to assess the potential of urinary metabolites sarcosine, uracil, and kynurenic acid as biomarkers for early diagnosis of PCa in three distinct individual categories and for prognosis of the progression and aggressiveness of the tumor. In order to study the above urine samples of individuals with radical prostatectomy, Ultrasonographically-Guided Prostatic Biopsy and controls were analyzed by a UPLC-MS/MS method able to detect and quantify the metabolites of interest including sarcosine, kynurenic acid and uracil. Multivariate and univariate analysis were further performed in order to find any correlation of the studied metabolites with the prognosis and the progression monitoring of prostate cancer.

## 2. Results

### 2.1. Characteristics of Study Population

The mean/average age (years) of participants was 65.3 ± 5.6 in group A (RP, Radical Prostatectomy), 67.7 ± 7.7 in group B (UGPB, Ultrasonographically-Guided Prostatic Biopsy), and 35.3 ± 6.8 in group C (control group). More information about the study groups and their clinical and histopathologic data are presented in Table 1. With regards to histopathological results, medians and ranges of PSA levels and Gleason Scores of patients in groups A + B are shown in Table 2 and Table 3.

### 2.2. Detection and Quantitation of Sarcoine, Uracil, and Kynurenic Acid in Urine Samples

**Figure 1:**
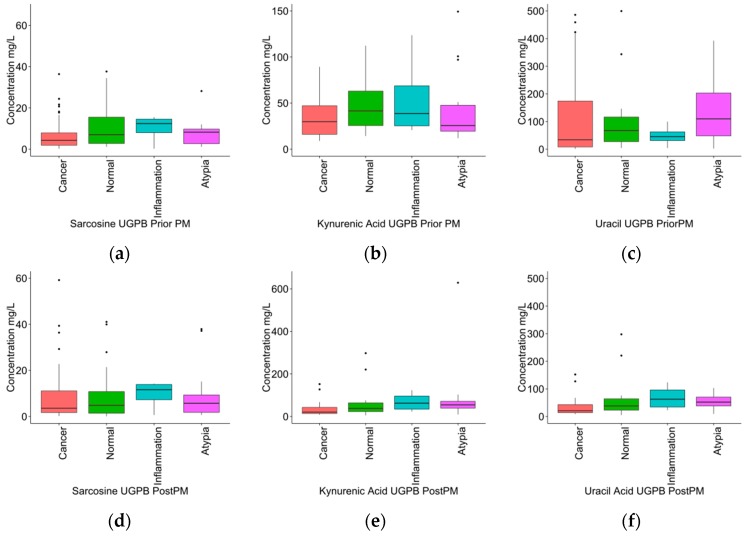
Box plots of potential markers (**a**) sarcosine, (**b**) kynurenic acid, and (**c**) uracil, in urine samples of patients in group B (UGBP) before prostatic massage and (**d**) sarcosine, (**e**) kynurenic acid, and (**f**) uracil. Boxes are drawn from the 25th to 75th percentiles in the concentration distribution.

Initially, UGPB group was studied in order to investigate any correlation between the levels of sarcosine, kynurenic acid, and uracil in urine samples with the histopathologic results such as positive or negative in cancer, atypia, and inflammation. LC-MS/MS results from prior and post prostatic massage (PM) were treated separately. Multivariate statistical analysis showed that the studied variables could not contribute to differentiation of the healthy participants from those with cancer, inflammation, or atypia. In Table 4, results from univariate statistical analysis of the urine metabolic data from pre- and post-PM can be observed. Although there were no statistical significant observations, in general sarcosine presented at higher concentration levels in case of inflammation for both pre- and post-PM categories and uracil levels were found increased in participants with normal results after UGPB only in samples that collected after PM. With regard to kynurenic acid, the average concentration in samples collected prior to PM was higher than the average concentration found in healthy subjects after UGPB; the case of post-PM urine was increased in patients with atypia. Box plots of sarcosine, uracil, and kynurenic acid in the UGPB group are shown in Figure 1.

In the second step, concentration levels of the studied metabolites in patients with positive histopathologic results after UGPB procedure and patients from group A (RP) were compared to the rest of the patients in group B, with histopathologic results negative in cancer, atypia, and inflammation. With regards to group B, only data from pre-PM urine samples were taken into consideration. In Table 5 results from non-parametric statistical analysis are presented. Again, differences observed between the concentration levels of metabolites in the studied groups did not present any statistical significance. However, sarcosine was still found at slightly higher levels in urine samples of patients with inflammation of the prostatic tissue, uracil decreased in urine samples of patients with atypia and overall, patients with normal histopathologic results had the highest concentrations of kynurenic acid. 

**Figure 2:**
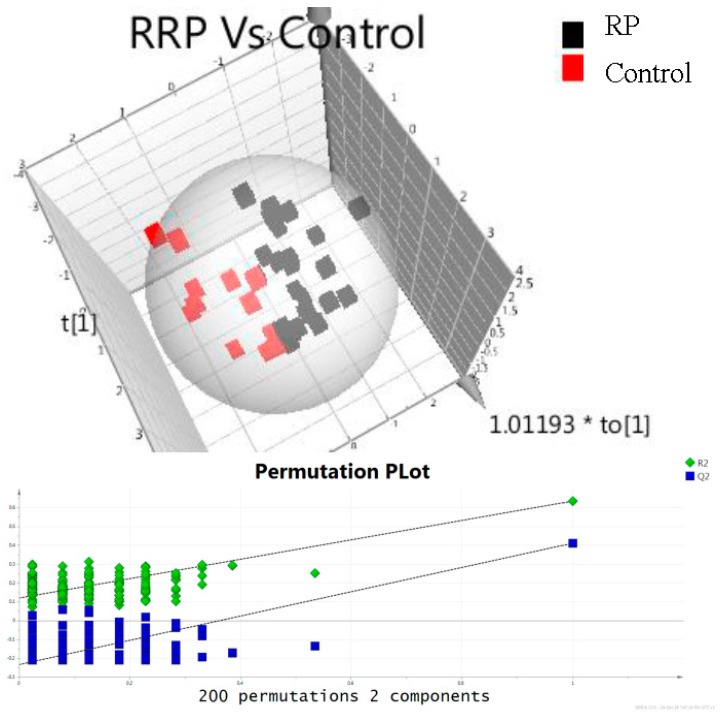
OPLS-DA score plot of data obtained from urine samples of group A and C with the validation plot group color: red—control; black—group A (RRP).

Finally, concentrations of metabolites in urine samples of patients in the three groups were examined (groups A, B, and C). Multivariate statistical analysis was performed in order to find any correlation between the studied metabolites and prostate cancer. In Figure 2, 3D Orthogonal Partial Least Square Discriminant analysis score plot of data obtained from samples of patients in group A and C is illustrated. Evaluation of the data set was performed on PLS-DA model using permutation test in order to guard against model overfitting. Results showed that the predictive value of the model is satisfactory. The data set provides robust models with high predictability as suggested by R2Y (cum) and Q2 values (0.634 and 0.410). In Table 6, results from univariate statistical analysis are shown. Participants with PCa undergoing RP presented highest sarcosine and uracil concentrations compare to those undergoing UGPB and controls (*p* = 0.01, Table 6). In the case of kynurenic acid, statistically significant higher concentration levels were observed in the control group compared to group A (*p* = 0.019) and B (*p* = 0.004). 

**Figure 3:**
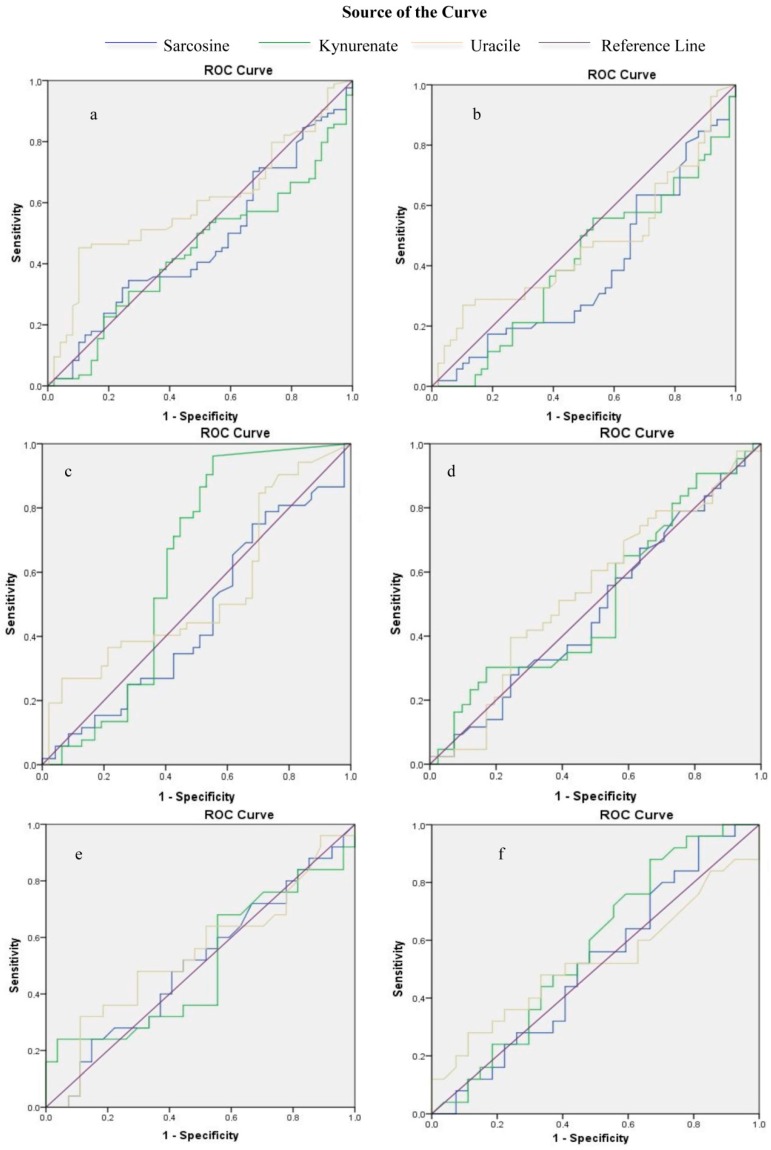
Receiver operating characteristic curves for sarcosine, kynurenic acid and uracil in urine (**a**) in relation to histopathological results; (**b**) in patients undergoing UGPB pre-PM; (**c**) in patients undergoing UGPB post-PM; (**d**) in relation to Gleason Scores; (**e**) in patients undergoing UGPB pre-PM; (**f**) in patients undergoing UGPB post-PM. Dependent variable (a)–(c): prostate cancer risk and (d)–(f): Gleason Score.

**Figure 4:**
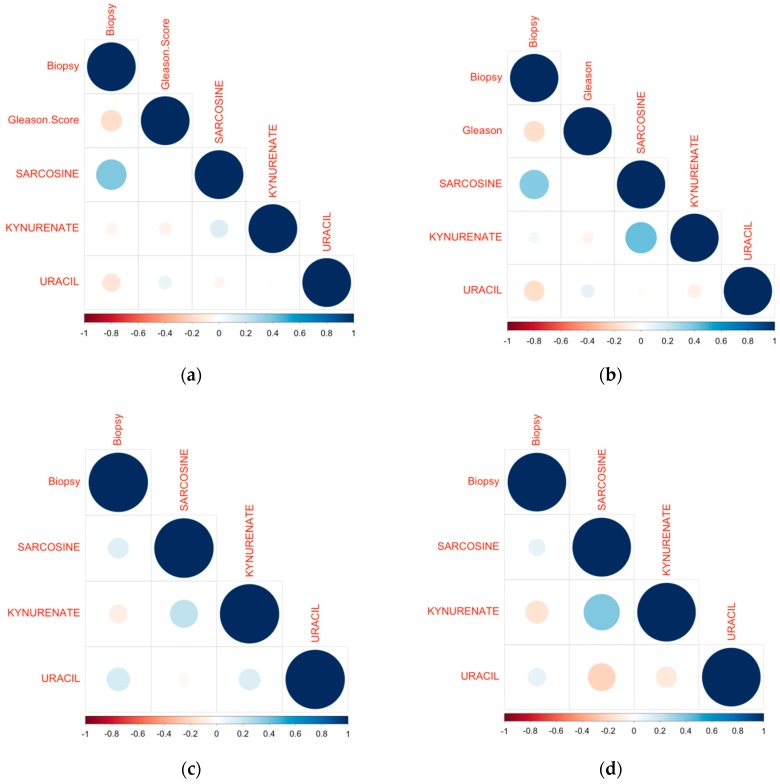
Pearson correlation heatmap of histopathologic data and measured metabolites in urine samples of group B patients (**a**) positive in UGBP, pre-PM; (**b**) positive in UGBP, post-PM; (**c**) negative in UGBP pre-PM; and (**d**) negative in UGBP post-PM patients.

In order to investigate the diagnostic value of endogenous metabolites, ROC analysis was performed. Biopsy results were used as a cut-off point between positive and negative results. Initially, analysis was performed for all participants and it was observed that both sarcosine (AUC: 0.47, *p* = 0.554) and kynurenic acid (AUC: 0.44, *p* = 0.251), had no diagnostic value. Uracil (AUC: 0.59, *p* = 0.066) showed the highest diagnostic value although not statistically significant (Figure 3a, Table S1 in Supplementary Materials). In patients undergoing UGPB none of the studied metabolites detected in pre-PM samples showed diagnostic potential (sarcosine: AUC: 0.38, *p* = 0.044, kynurenic acid: AUC: 0.41, *p* = 0.128, uracil AUC: 0.47, *p* = 0.594). Kynurenic acid in post-PM urine samples presented the highest significant diagnostic value (AUC: 0.62, *p* = 0.041) compared to sarcosine and uracil (sarcosine: AUC: 0.46, *p* = 0.473, uracil: AUC: 0.54, *p* = 0.492) (Figure 3b,c, Tables S2 and S3 in Supplementary Material). Pearson correlation was also performed on data from patients in group B found positive in prostate cancer. In Figure 4a,b (Pearson correlation heatmap) it can be observed that sarcosine correlates positively with biopsy results in both pre- and post-PM cases. Figure 4c,d presents the Pearson correlation heatmap with regard to patients from group B found negative in prostate cancer. For both pre- and post-PM cases, uracil and sarcosine correlate positively, while kynurenic acid correlates negatively with biopsy results. ROC curves were also used to investigate the diagnostic value of endogenous metabolites in the monitoring of PCa progression. Gleason score was used as a cut-off point between high and low aggression. Again, results from urine samples of all participants did not show any predictive value, for sarcosine (AUC: 0.48, *p* = 0.819), kynurenic acid (AUC: 0.51, *p* = 0.858), and uracil (AUC: 0.54, *p* = 0.525) (statistically non-significant, Figure 3d, Table S4 in Supplementary Materials). Low diagnostic value was observed for uracil in pre-PM urine samples of patients undergoing UGPB (uracil: AUC: 0.54, *p* = 0.595, sarcosine: AUC: 0.51, *p* = 0.927, kynurenic acid: AUC: 0.5, *p* = 0.985), while kynurenic acid showed a slightly increased diagnostic value in post-PM urine samples (kynurenic acid: AUC: 0.57, *p* = 0.36, sarcosine: AUC: 0.52, *p* = 0.84, uracil: AUC: 0.52, *p* = 0.777) (Figure 3e,f, Tables S5 and S6 in Supplementary Materials), however both findings were not statistically significant.

Additionally, Pearson correlation was performed in order to assess any correlation between the detected metabolites in patients with prostate cancer from group B and Gleason score, and only kynurenic acid was found to slightly correlate negatively (−0.2) with Gleason score. 

## 3. Discussion

Prostate cancer is a significant cause of morbidity and mortality in men globally and the currently used diagnostic modalities cannot identify all cancer cases or predict their clinical evolution. Metabolomics try to address these limitations and, if possible, to provide better tools for the selection of the optimal treatment and monitoring of disease progression. The present pilot study is an attempt to investigate whether sarcosine in urine is a suitable alternative biomarker for PCa detection and progression monitoring [5,12]. Uracil and kynurenic acid were found as promising biomarkers in urine samples of prostate cancer patients, when urine is collected before and after prostatic massage [5]. Urine is abundantly available, easily and noninvasively collected, and can be used to detect either exfoliated cancer cells expressing specific biomarkers or secreted products—such as proteins, nucleobases, or nucleic acids—that may differ from those found in healthy population. It is therefore the ideal body fluid for metabolic profiling in cases of prostate cancer [16].

Sarcosine is a naturally occurring derivative of glycine, formed by the enzymes glycine *N*-methyl transferase (GNMT) or dimethylglycine dehydrogenase (DMGDH), and converted back into glycine via sarcosine dehydrogenase (SARDH). Sarcosine is not specific to prostatic tissue, and it plays a significant role in methyl balance as an intermediate compound in the metabolism of choline and methionine [17,18]. In 2009, a study reported the potential role of sarcosine as a biomarker for PCa detection and prognosis [5]. In a group of 110 patients, sarcosine was found significantly higher in urine sediments and supernatants in men with PCa compared to men with negative biopsies. The mean PSA values in this study were not equally distributed between PCa patients and biopsy negative controls [5]. Sarcosine, uracil, kynurenine, and other metabolites were reported as being significantly elevated upon disease progression, from benign to PCa to metastatic disease [5]. Varambally et al. reported in 2002 that overexpression of histone-lysine *N*-methyltransferase (EZH2) in benign cells could lead to cell invasion and neoplastic progression [19]. Because sarcosine levels were also found to be associated with cell invasiveness, Sreekumar et al. studied the association of sarcosine with EZH2 and found that overexpression of EZH2 in benign cells increased sarcosine levels, while knockdown of EZH2 in cancerous cells decreased the amount of the metabolite [5]. Sarcosine was reported to be directly related to the process of cancer invasion and the enzymes that regulate sarcosine levels could be potential targets for modulation of prostate cancer invasion [5]. These data prompted Baum et al. to declare that sarcosine is not only a novel and predictive biomarker but a key element of a potentially promising target pathway for the treatment and control of prostate cancer development [20].

In contrast, another study—performed in 139 men with more comparable PSA biopsy values between PCa and non-PCa patients—found significantly lower sarcosine values in PCa patients compared to non-PCa patients and no significant difference between healthy men and PCa patients [12]. Also, % fPSA had a significantly larger AUC than sarcosine (0.81 vs. 0.63; *p* = 0.012), and PSA was equal to sarcosine (0.64 vs. 0.63; *p* = 0.93). In this study, sarcosine was measured by gas chromatography–mass spectrometry and sarcosine values were normalized to urine creatinine [12]. Another group measured sarcosine levels in the supernatant of post-DRE urine of 17 PCa patients and compared them with the values in biopsy-negative patients [12]. They did not find any statistically significant difference between the two groups, although they observed increased sarcosine levels in samples from healthy participants. A correlation between sarcosine levels and total PSA or PCa antigen 3 was not shown [12]. Using the isotope dilution GC/MS approach with microwave-assisted derivatization, Wu et al. did not find any statistical difference in urinary sarcosine levels between PCa patients and the control group [21]. Yet another group evaluated sarcosine levels in urine supernatants and sediments and used PCa antigen 3 (PCA3) and % fPSA as comparators [22]. Regardless of the specimen type, sarcosine was significantly higher in PCa patients than in controls, but there was no correlation with the Gleason score or clinical stage [22].

Kynurenic acid is an NMDA antagonist produced from kynurenine, a derivative of tryptophan [23]. Tryptophan is catalyzed by two distinct enzymes, tryptophan 2,3-dioxygenase (TDO), and indoleamine 2,3-dioxygenase (IDO). Both TDO and IDO lead to oxidative cleavage of tryptophan pyrrole ring resulting in formation of kynurenine, which is subsequently converted to kynurenic acid by kynurenine aminotransferase (KAT). The ratio of kynurenine/tryptophan in serum was investigated as a potential marker for detecting prostate cancer [24]. Data on the potential use of uracil as a biomarker for prostate cancer detection and progression monitoring are limited [5,14].

In the present study, the median sarcosine and kynurenic acid values were found to be lower in PCa patients than in participants without malignancy, in accordance to previously published results [12,14]. The opposite (higher values in PCa patients) was observed for uracil. Sarcosine, uracil, and kynurenic acid values were not associated with age or tumor grade (Gleason score <7 vs. ≥7). ROC curve analyses showed that the differentiation of PCa patients and participants without malignancy was not enhanced by sarcosine or uracil measurements in comparison with total PSA, but kynurenic acid was found to improve detection of prostate cancer when measured in urine samples collected after prostatic massage. Prostatic massage is performed in order to obtain a prostatic fluid sample through urine collection and there is speculation that this intervention causes clinical significant changes in excretions of metabolites that will end up in the post prostatic massage samples, thus will be detected in urine samples. This result contradicts previously published data but this can be explained due to differences in study design including sample collection [14]. However, the need to collect urine after prostatic massage, in order to use kynurenic acid for diagnostic purposes as an alternative or additional biomarker, might discourage at least some patients from attending population-based screening for prostate cancer due to anticipated pain or discomfort [25]. This might diminish the advantages of such an approach.

The weaknesses of this study are the large number of PCa patients compared to the number of healthy participants and the lack of data regarding tumor staging. It is difficult and possibly unethical to subject healthy individuals to invasive clinical examinations and/or procedures, thus the control group was recruited only from individuals presenting for other etiologies and these patients usually are much younger than those presenting prostate cancer.

On the other hand, advantages of this study are the nature of a prospective study design with well-defined study groups, adequate sample sizes, and more comparable PSA values among participants.

In summary, the data presented here show that urinary sarcosine and uracil are not found as suitable biomarkers for the detection of prostate cancer, in contrast to kynurenic acid, when it is measured in urine collected after prostatic massage. None of the three metabolites studied can be reliably used for monitoring of disease progression. As it was referred above, results from the present study contradict previous publications but we acknowledge that there are significant methodological differences and we attribute them to differences in clinical design and recruitment of study population. Variations in the results of investigations on the potential of sarcosine and related metabolites as PCa biomarkers have been attributed to variations and the lack of harmonization in study design, inclusion/exclusion criteria for subject recruitment, as well as in the sampling process.

## 4. Materials and Methods

### 4.1. Reagents and Materials

LC/MS grade Acetonitrile (ACN) was abtained from Carlo Erba (Van de Remil, Paris, France). Distilled Water (18.2 MΩ) for chromatographic separation was purified in Milli-Q device (Millipore, Merch Darmstadt, Germany). Ammonium formate (NH_4_HCO_2_) was purchased from Sigma Aldrich (Gillingham, UK). The standards were of analytical or higher grade and for this study obtained from various vendors. 

### 4.2. Samples and Sample Preparation

Patients were recruited from an outpatient clinic at a tertiary University Hospital Department (Aristotle University Urology Clinic). Three groups of patients were included in this study. group Α (RP): 32 patients undergoing radical prostatectomy (RP) for verified clinically localized prostate cancer. Urine samples were collected preoperatively; group Β (UGPB): 101 patients with increased PSA levels undergoing transrectal ultrasonographically-guided prostatic biopsy. Urine samples were collected before and after prostatic massage (pre-/post-PM). Control group—group C: 15 healthy volunteers (Table 1). Urine samples of second morning void midstream were collected from all participants and PM was performed via DRE by administration of three strokes per lobe. Urine samples were stored at −80 °C after post-centrifugation (each sample centrifuged at 1500× *g*, for 10 min, at 4 °C) until further analysis. Biopsies were evaluated by an experienced board certified clinical pathologist. All participants gave appropriate oral and written consent prior to sample collection and the study was approved by the ethics committee of the hospital.

For LC-MS/MS analysis a volume of 100 μL of sample was diluted with 100 μL of MeOH. The samples were subsequently vortex mixed (1 min) and centrifuged for 10 min (7000 *g*) to remove particulate matter and macromolecules. 50 μL of supernatant was diluted with 100 μL of MeCN and transferred to LC/MS vial and loaded on the autosampler tray, which was maintained at 10 °C for the duration of analysis. For the calibration curve, since there is no available free analyte matrix, urine samples of healthy population were diluted (1:1 *v/v*) with water, subsequently vortexed, and centrifuged 7000 rpm for 10 min. Supernatants were diluted with MeOH (1:2 *v/v*) vortexed and centrifuged at 7000 rpm for 10 min. That process resulted in a diluted matrix containing minimum concentration of endogenous compounds. Finally, 50 μL of supernatant was diluted with 90 μL of MeCN and 10 μL of standard (eight standard points).

### 4.3. Determination of Sarcosine, Uracil, and Kynurenic Acid by LC-MS/MS

Analysis of urine samples was performed on an Ultra High-Performance Liquid Chromatography coupled with a mass spectrometry Xevo TQD system (Waters Corporation, Milford, MA, USA). Separation was performed on a ACQUITY UPLC™ BEH AMIDE column 1.7 μm, 2.1 mm × 150 mm (Waters Corporation, Milford, MA, USA) suitable for polar metabolites. For method development, modification of conditions from a previously reported HILIC-MS/MS method was performed [26]. Sarcosine, uracil, and kynurenic acid were detected using Multiple Reaction Monitoring (MRM) mode in a single injection of 15.5 min. The MRM transitions for the three metabolites were set as follows: sarcosine *m*/*z* 90–44, CV = 20 V, CE = 8 V; uracil *m*/*z* 113–70, CV = 40 V, CE = 15 V; and kynurenic acid *m*/*z* 190–172, CV = 32 V, CE = 12 V. For chromatographic separation the mobile phase was a mixture of (A) ACN:H_2_O, 95:5 *v/v* and (B) H_2_O:ACN, 70:30 *v/v* both with final ammonium formate buffer concentration of 10 Mm and elution was performed with a gradient program started with 100% A, then rising to 15% B linearly over the next 2 min, finally reaching 40% B over 2 min and returning to initial conditions over 5 min. The column was equilibrated for 6 min in the initial conditions. Flow rate was 0.5 mL/min. For the quantitation of detected compounds, an eight point calibration curve was running at the beginning and the end of each analytical set, and the mean calibration curve was used for the semi-quantitation of the analytes. The correlation coefficient R^2^ resulted from calibration curve was 0.998 for sarcosine, 0.996 for uracil, and 0.996 for kynurenic acid, indicating good linearity of the method over a wide range of concentrations (1 μg/L–1 mg/L). The quantitation limit (Limit of Quantitation, LOQ) was 1 μg/L and the detection limit (Limit of Detection, LOD) was 0.3 μg/L for all the compounds and was calculated after three replicate injections of each standard (LOD = 3 * SD, LOQ = 3 * LOD). The accuracy of the method (%) was calculated based on protocols for metabolic profiling described in a series of publications from our group [27,28,29]. Replicate injections of urine samples spiked at different concentrations were performed and accuracy ranged for sarcosine from 90% to 95%, for uracil from 89% to 96%, and for kynurenic acid from 90% to 98% while relative standard deviation (RSD, %) was found to range from 4.2% to 11% for sarcosine, from 2.7% to 10% for uracil, and from 4.2% to 10.5% for kynurenic acid. Recovery (%) was assessed after spiking urine samples with a standard before and after extraction and it was found to be 112% for sarcosine, 121.4% for uracil, and 113.7%. for kynurenic acid. Chromatographic peaks of the studied metabolites in diluted urine samples can be observed in Supplementary Figure S1. 

Actual concentrations of the detected metabolites in urine samples study population were used for further statistical analysis. Urinary concentrations of metabolites were not normalized to urinary creatinine since that could result in an underestimation or overestimation of the biomarker excretion rate depending on the clinical context. It has been reported that quantification of urinary metabolites requires the collection of timed urine specimens to estimate the actual excretion rate, provided that the biomarker is stable over the period of collection. This idea must be balanced, however, against practical considerations [30]. Some studies suggest that normalization to urinary creatinine may be inappropriate at times, as urinary creatinine excretion rate may vary greatly, depending on the situation. 

### 4.4. Statistical Analysis

Statistical calculations were performed with SPSS Statistics 22 (SPSS Software, Chicago, IL, USA). Continuous variables following/not following normal distribution were expressed as averages (mean) and standard deviations (SD)/medians (median) and ranges (range: maximum–minimum), respectively. Dichotomous and categorical variables were expressed as frequencies and percentages. Control of the regularity of the variables was performed using the Shapiro–Wilk test for variables with less than 50 cases and the Kolmogorov–Smirnov test for variables with more than 50 cases. The Mann–Whitney U-test was used for comparing means between two independent samples of continuous variables not normally distributed and the Kruskal–Wallis test was used for comparing means between more than two independent samples.

Multivariate statistical analysis was performed with Simca P13 software (Umetrics, Upsala, Sweden). For Pearson correlation, the corrplot script package was used in R.

Receiver Operating Characteristic (ROC) curves analyses were performed in order to evaluate the diagnostic value of endogenous metabolites for the prognosis of prostate cancer and the predictive value with regards to the aggressiveness of prostate cancer according to Gleason score. Biopsy results were used as a cut-off point between positive and negative results to predict prostate cancer. Gleason score values were used as the boundary between high and low aggressiveness (Gleason score values ≥7 were considered highly aggressive and labeled High Grade, Gleason score values <7 were considered poorly aggressive and labeled Low Grade). A *p* value <0.05 (two-sided) was considered statistically significant.

## Figures and Tables

**Table 1:** Patient groups, clinical and histopathologic characteristics. Values are presented as means/SDs, medians/ranges or percentages.

Data		Subject Group		
		A (RP)	B (UGBP)	C (Control)
Number		32	101	15
% of Total Study Population		21.6	68.2	10.2
Age		65.3 ± 5.6	67.7 ± 7.7	35.3 ± 6.8
BMI		27.8 ± 4.1	27.1 ± 3.6	25.9 ± 4.9
Prostate Cancer Positive		32	52	-
Gleason Score Low		14	27	-
Gleason Score High		18	74	-
Histopathology Results	PCa	-	52	-
	Inflammation	-	4	-
	Atypia	-	15	-
	Normal	-	30	-
DRE	Pathologic	-	38	-
	Normal	-	36	-

Tumor Grade according to Gleason Score <7: Low Grade, Gleason Score ≥7: High Grade.

**Table 2:** Median serum PSA levels in relation to histopathologic results—group A + B. Values are presented as medians/ranges.

Data	PSA		*p*
Histopathologic Result	Median	Range	
PCa	8.3	2.6–146.1	-
Inflammation	6.5	6.1–7.1	-
Atypia	8.3	4.5–20.3	0.102
Normal	6.5	3.6–27.7	-

PSA: prostatic specific antigen.

**Table 3:** Comparison of median PSA levels in relation to Gleason Score—group A + B. Values are presented as medians/ranges.

Data	PSA		*p*
Gleason Score	Median	Range	
Low Grade	7.5	3.8–23.6	0.456
High Grade	7.8	2.6–146	

PCa: prostate cancer; PSA: prostatic specific antigen; Tumor Grade according to Gleason Score <7: Low Grade; Gleason Score >7: High Grade.

**Table 4:** Endogenous metabolites in urine according to histopathologic results—group A + B. Values are presented as medians/ranges in mg/L.

Histopathologic Results					*p*
Metabolites	PCa	Inflammation	Atypia	Normal	
Sarcosine	5.6 (0.25–65.1)	12.5 (0.3–15.5)	7.1 (1.2–28.2)	10.9 (1.2–37.7)	0.842
Uracil	106.9 (0–2171.7)	45.4 (4.1–99.6)	95.5 (0–392.7)	60.4 (4.7–499.4)	0.297
Kynurenic acid	29.9 (1.80–125.4)	38.7 (20.7–123.7)	25.5 (10.1–149.3)	41.6 (12.5–112.4)	0.345

pre-PM: before prostatic massage; post-PM: after prostatic massage; PCa: prostate cancer.

**Table 5:** Endogenous metabolites according to histopathologic results—group B. Values are presented as medians/ranges in μg/L.

Histopathologic Results					*p*
	Pca	Inflammation	Atypia	Normal	
Sarcosine pre-PM	4.3 (0.25–36.4)	12.5 (0.3–15.5)	7.1 (1.2–28.2)	7.1 (1.2–37.7)	0.219
Sarcosine post-PM	3.4 (0.25–59.2)	9.5 (0.6-13.8)	3.7 (0.6–37.9)	5.2 (0.1–41.1)	0.908
Uracil pre-PM	24.6 (0–485.9)	45.4 (4.1–99.6)	95.5 (0–392.7)	60.4 (4.7–499.4)	0.864
Uracil post-PM	19.9 (0–448.4)	41.1 (15.2–72.7)	3.8 (0–267.3)	63.6 (0–500.6)	0.132
Kyn. acid pre-PM	29.9 (9.1–89.4)	38.7 (20.7–123.7)	25.5 (10.1–149.3)	41.6 (12.5–112.4)	0.181
Kyn. acid post-PM	20.4 (0–152.3)	0 (0–38.2)	24.7 (0–628.7)	9.6 (0–297.8)	0.104

group B: patients undergoing UGPB; pre-PM: before prostatic massage; post-PM: after prostatic massage; PCa: prostate cancer; Kyn. acid: kynurenic acid.

**Table 6:** Endogenous metabolites in urine according to intervention—group A + B + C. Values are presented as medians/ranges in μg/L.

Metabolites	Intervention			*p*
	RP (*n* = 32)	UGPB (*n* = 101)	None (*n* = 15)	
Sarcosine	13.2 (0.4–65.1)	5.8 (0.3–37.7)	12.7 (2.1–34.5)	0.011
Uracil	222.1 (22.6–2171.7)	42.8 (0–499.4)	41.1 (10.1–111.1)	<0.001
Kyn. acid	33.9 (1.8–125.4)	30.1 (9.1–149.3)	67.4 (20.5–146.4)	0.019

## Supplementary Materials

The following are available online at www.mdpi.com/2218-1989/7/1/9/s1, Figure S1: Chromatographic peaks of (a) sarcosine, (b) uracil and (c) kynurenic acid in urine samples; Data regarding AUCs, SEs, p and CIs from Receiver operating characteristic analysis are presented in Tables S1–6.

## Abbreviations

PCa	Prostate Cancer
DRE	Digital Rectal Examination
PSA	Prostate Specific Antigen
Pre-/Post-PM	Before-/After Prostatic Massage
UGPB	Ultrasonographically-Guided Prostatic Biopsy
RP	Radical Prostatectomy
UPLC-MS/MS	Ultrahigh Pressure Liquid Chromatography coupled with Mass Spectrometry
SD	Standard Deviation
ROC	Receiver Operating Characteristic
SE	Standard Error
CI	Confidence Interval
EZH2	Histone-lysine *N*-methyltransferase
CV	Cone Voltage
CE	Collision Energy
