# Supplementary material for: The Role of Sarcosine, Uracil, and Kynurenic Acid Metabolism in Urine for Diagnosis and Progression Monitoring of Prostate Cancer
Source: Metabolites. 2017 Feb 23;7(1):9. doi: 10.3390/metabo7010009 (PMC5372212; doi:10.3390/metabo7010009)
Supplement: Supplementary file 1 [file metabolites-07-00009-s001.pdf]

Supplamentary Materials: The Role of Sarcosine, Uracil, and Kynurenic Acid Metabolism in Urine for Diagnosis and Progression Monitoring of Prostate Cancer

Georgios Gkotsos, Christina Virgiliou, Ioanna Lagoudaki, Chrysanthi Sardeli, Nikolaos Raikos, Georgios Theodoridis and Georgios Dimitriadis

Data regarding AUCs, SEs, *p* and CIs from Receiver operating characteristic analysis are presented in Tables S1–6

Table S1

Endogenous metabolites	AUC	SE	*p*	95% CI
**Sarcosine**	0,47	0,052	0,554	0,37-0,57
**Kynurenic acid**	0,44	0,05	0,251	0,34-0,54
**Uracil**	0,59	0,05	0,066	0,49-0,69

Table S2

Endogenous metabolites	AUC	SE	*p*	95% CI
**Sarcosine**	0,38	0,057	0,044	0,27-0,49
**Kynurenic acid**	0,41	0,057	0,128	0,31-0,52
**Uracil**	0,47	0,059	0,594	0,35-0,58

Table S3

Endogenous metabolites	AUC	SE	*p*	95% CI
**Sarcosine**	0,46	0,059	0,473	0,34-0,57
**Kynurenic acid**	0,62	0,062	0,041	0,49-0,74
**Uracil**	0,54	0,059	0,492	0,42-0,66

Table S4

Endogenous metabolites	AUC	SE	*p*	95% CI
**Sarcosine**	0,48	0,064	0,819	0,36-0,61
**Kynurenic acid**	0,51	0,064	0,858	0,38-0,63
**Uracil**	0,54	0,064	0,525	0,42-0,67

Table S5				
**Endogenous metabolites**	**AUC**	**SE**	***p***	**95% CI**
**Sarcosine**	0,51	0,081	0,927	0,35-0,67
**Kynurenic acid**	0,5	0,083	0,985	0,34-0,66
**Uracil**	0,54	0,082	0,595	0,38-0,71

Table S6				
**Endogenous metabolites**	**AUC**	**SE**	***p***	**95% CI**
**Sarcosine**	0,52	0,081	0,84	0,36-0,68
**Kynurenic acid**	0,57	0,08	0,36	0,42-0,73
**Uracil**	0,52	0,083	0,777	0,36-0,69

Supplementary Figure S1

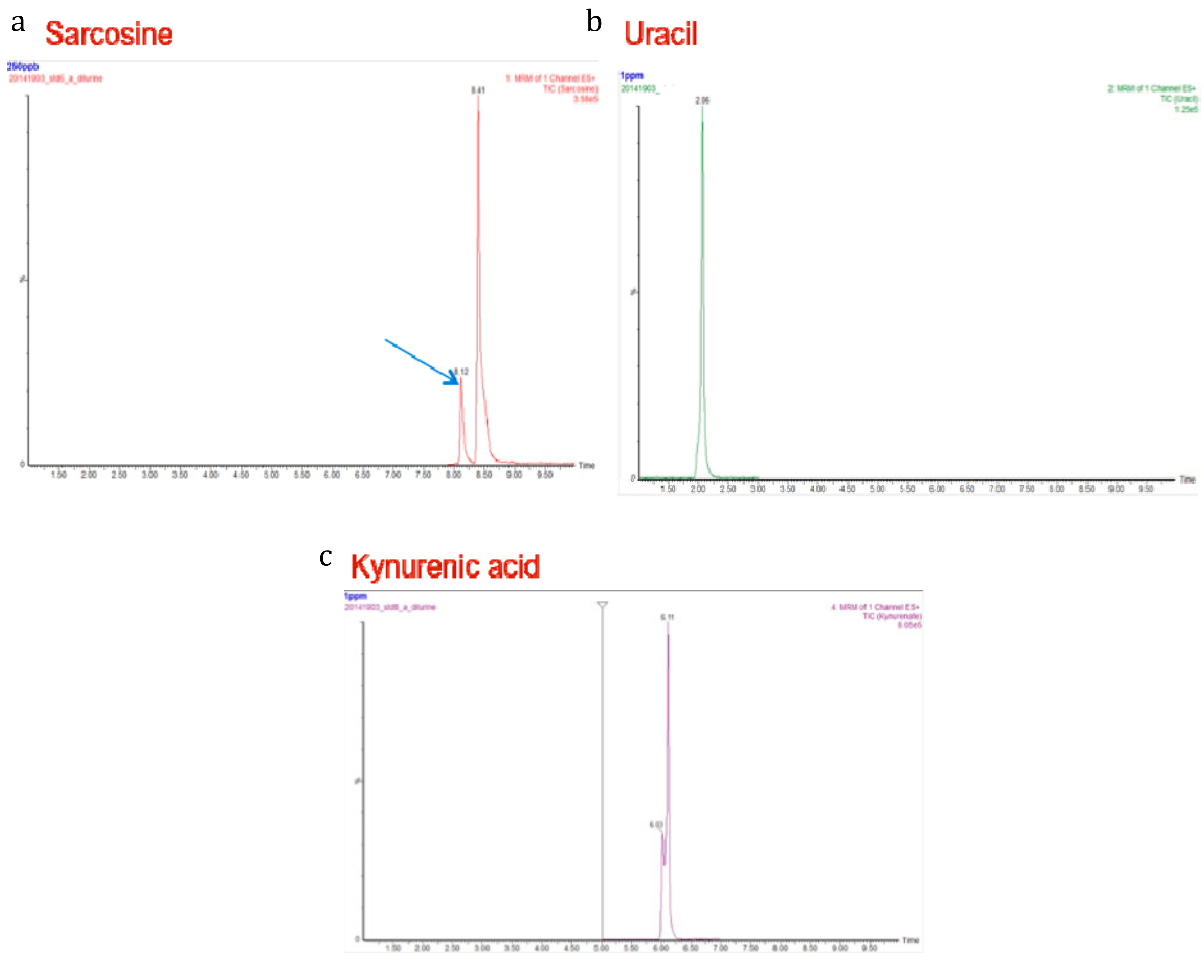

Figure S1: Chromatographic peaks of a) sarcosine, b)uracil and c)kynurenic acid in urine samples.
